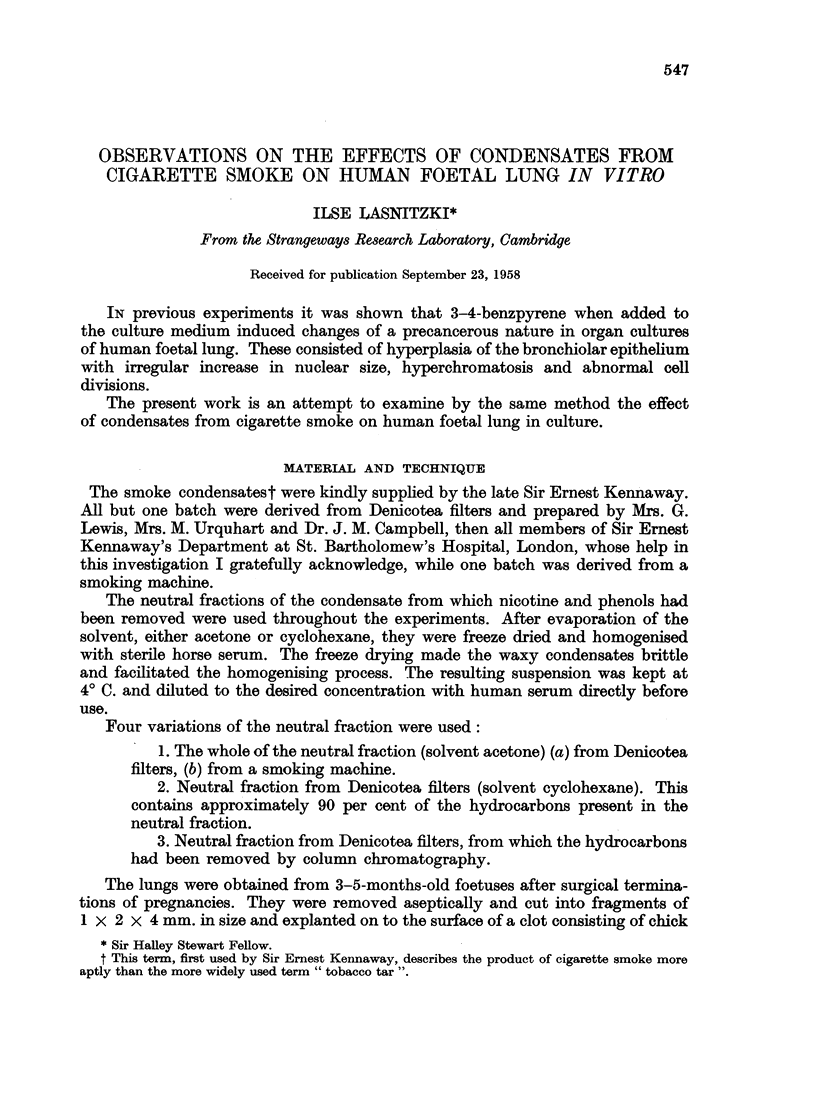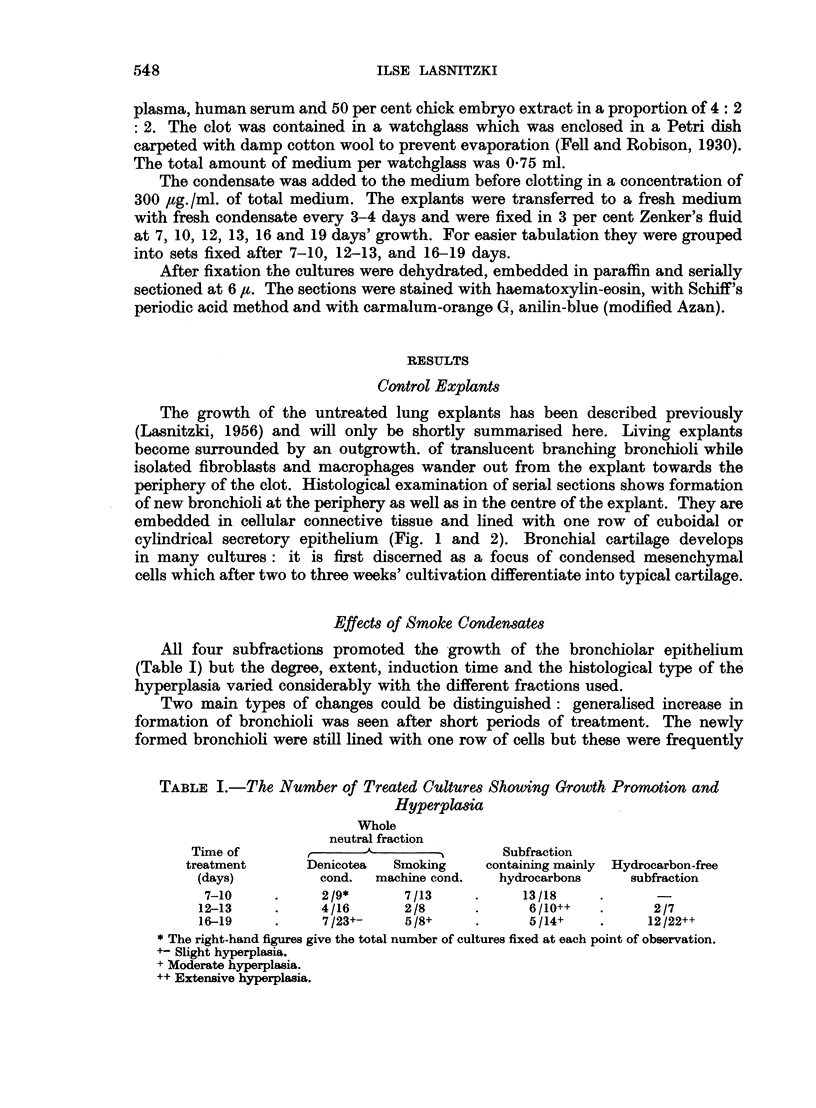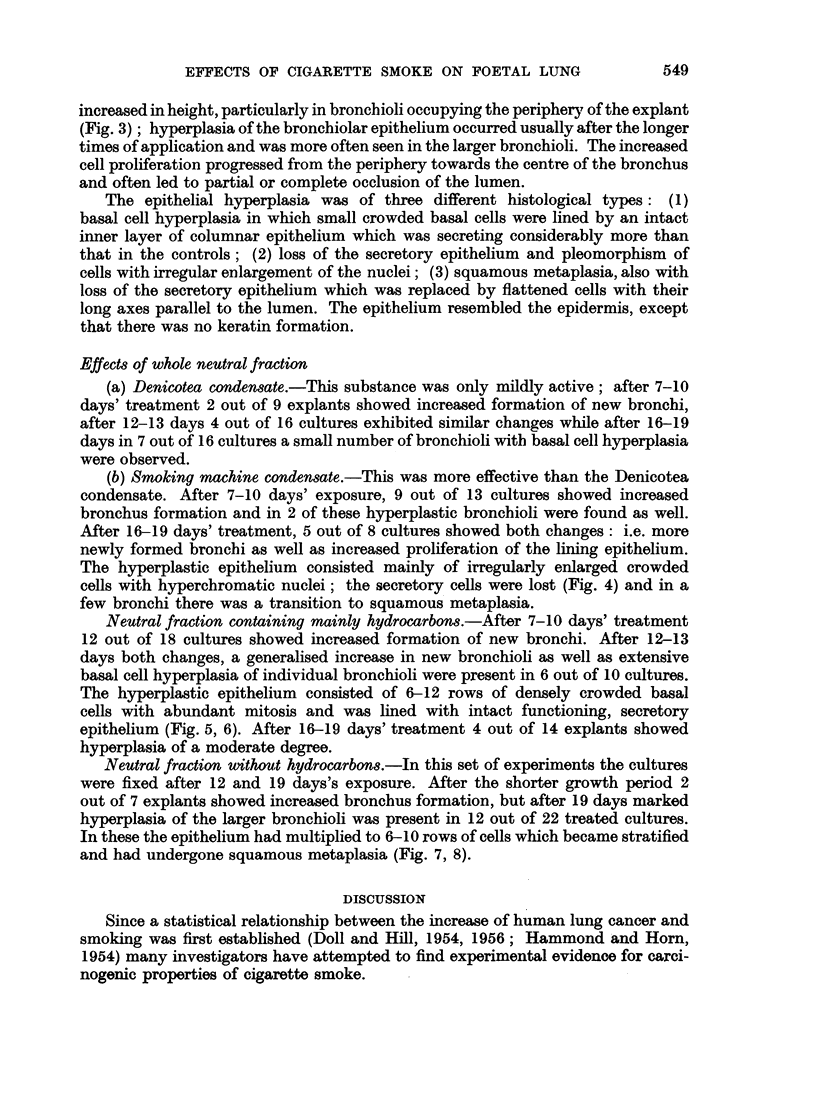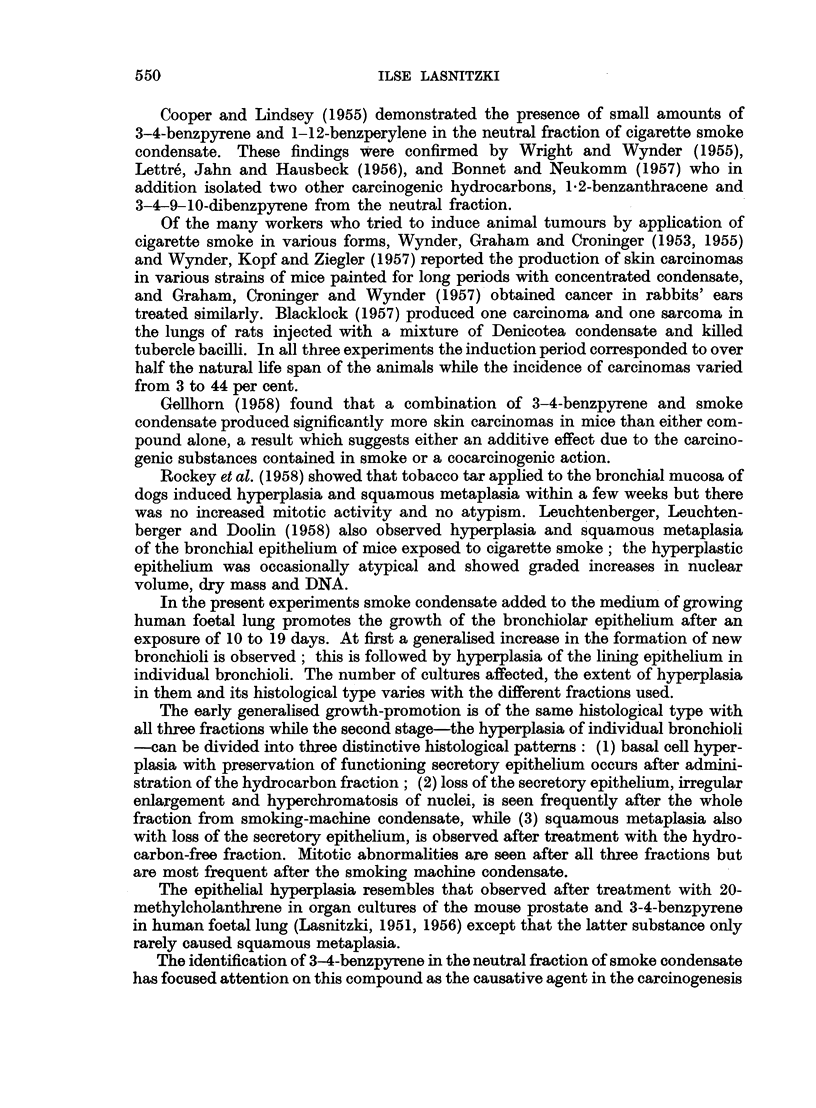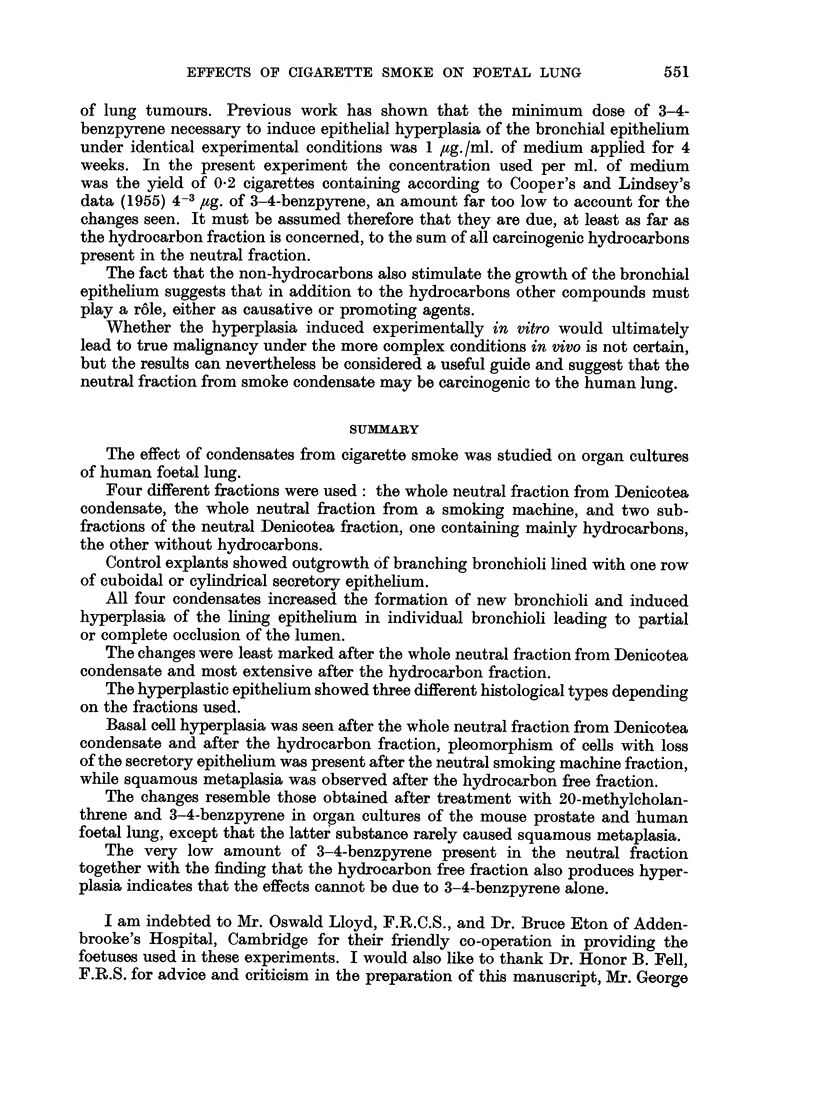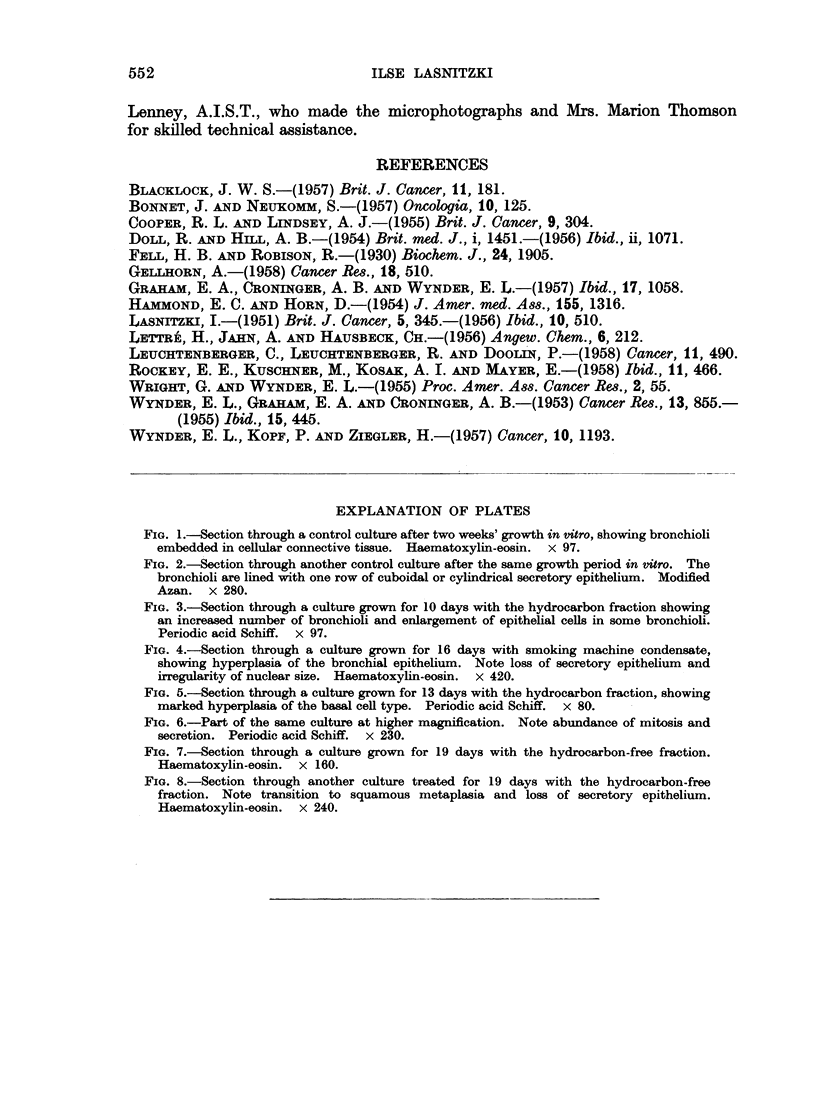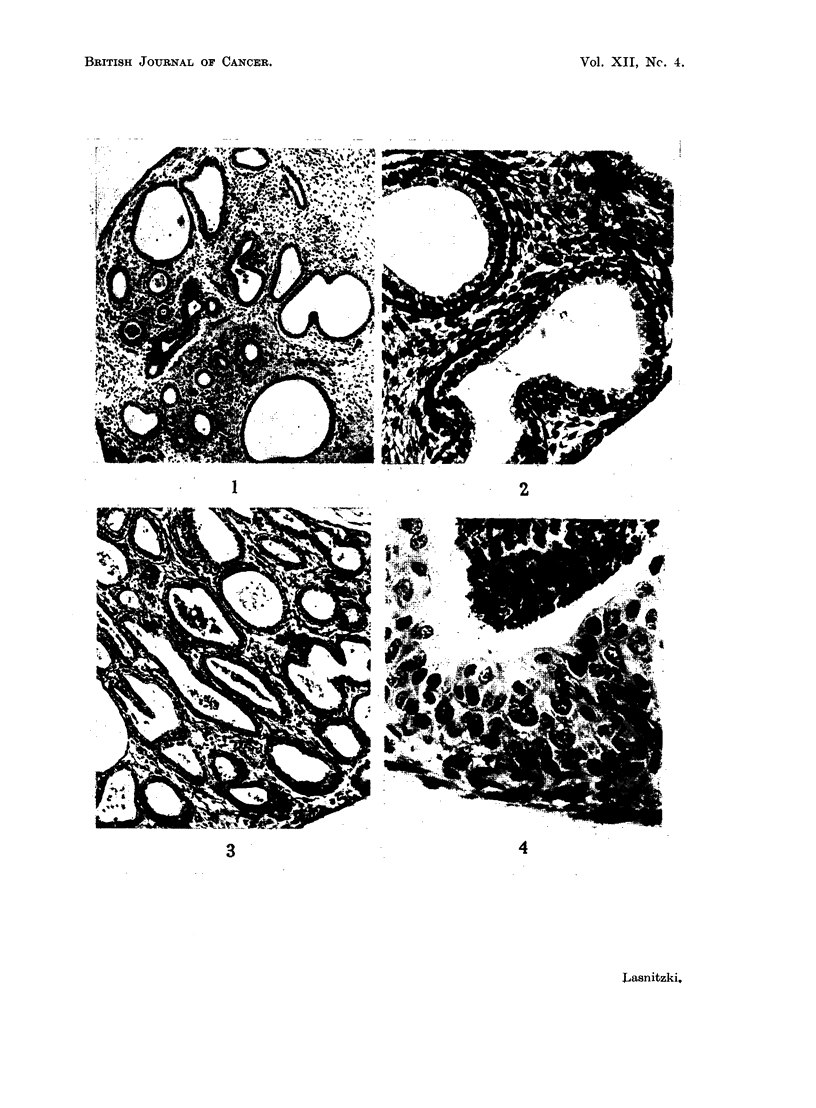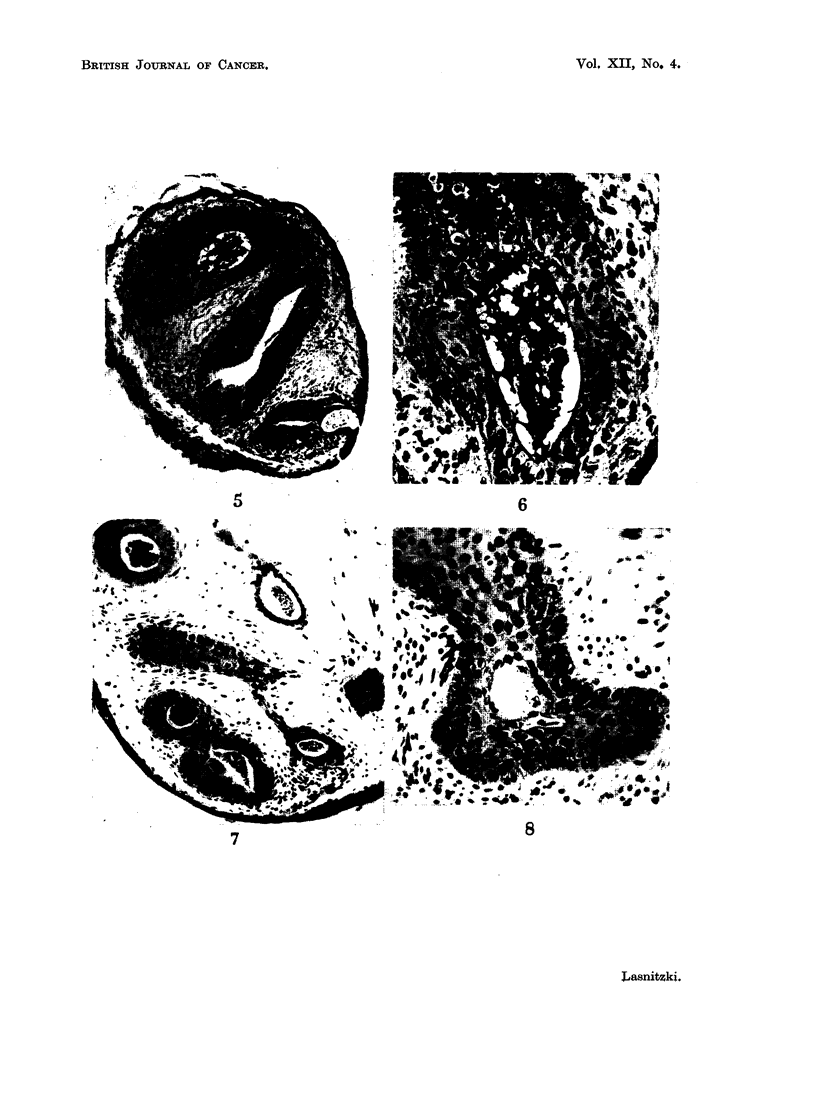# Observations on the Effects of Condensates from Cigarette Smoke on Human Foetal Lung In Vitro

**DOI:** 10.1038/bjc.1958.63

**Published:** 1958-12

**Authors:** Ilse Lasnitzki

## Abstract

**Images:**


### OBSERVATIONS ON THE EFFECTS OF CONDENSATES FROM CIGARETTE SMOKE ON HUMAN FOETAL LUNG *IN VITRO*

ILSE LASNITZKI*

*From the Strangeways Research Laboratory, Cambridge*

Received for publication September 23, 1958

In previous experiments it was shown that 3–4-benzpyrene when added to the culture medium induced changes of a precancerous nature in organ cultures of human foetal lung. These consisted of hyperplasia of the bronchiolar epithelium with irregular increase in nuclear size, hyperchromatosis and abnormal cell divisions.

The present work is an attempt to examine by the same method the effect of condensates from cigarette smoke on human foetal lung in culture.

### MATERIAL AND TECHNIQUE

The smoke condensates† were kindly supplied by the late Sir Ernest Kennaway. All but one batch were derived from Denicotea filters and prepared by Mrs. G. Lewis, Mrs. M. Urquhart and Dr. J. M. Campbell, then all members of Sir Ernest Kennaway's Department at St. Bartholomew's Hospital, London, whose help in this investigation I gratefully acknowledge, while one batch was derived from a smoking machine.

The neutral fractions of the condensate from which nicotine and phenols had been removed were used throughout the experiments. After evaporation of the solvent, either acetone or cyclohexane, they were freeze dried and homogenised with sterile horse serum. The freeze drying made the waxy condensates brittle and facilitated the homogenising process. The resulting suspension was kept at 4° C. and diluted to the desired concentration with human serum directly before use.

Four variations of the neutral fraction were used :

1. The whole of the neutral fraction (solvent acetone) (*a*) from Denicotea filters, (*b*) from a smoking machine.
2. Neutral fraction from Denicotea filters (solvent cyclohexane). This contains approximately 90 per cent of the hydrocarbons present in the neutral fraction.
3. Neutral fraction from Denicotea filters, from which the hydrocarbons had been removed by column chromatography.

The lungs were obtained from 3–5-months-old foetuses after surgical terminations of pregnancies. They were removed aseptically and cut into fragments of 1 × 2 × 4 mm. in size and explanted on to the surface of a clot consisting of chick

* Sir Halley Stewart Fellow.

† This term, first used by Sir Ernest Kennaway, describes the product of cigarette smoke more aptly than the more widely used term " tobacco tar ".

plasma, human serum and 50 per cent chick embryo extract in a proportion of 4 : 2 : 2. The clot was contained in a watchglass which was enclosed in a Petri dish carpeted with damp cotton wool to prevent evaporation (Fell and Robison, 1930). The total amount of medium per watchglass was 0·75 ml.

The condensate was added to the medium before clotting in a concentration of 300 $\mu$g./ml. of total medium. The explants were transferred to a fresh medium with fresh condensate every 3–4 days and were fixed in 3 per cent Zenker's fluid at 7, 10, 12, 13, 16 and 19 days' growth. For easier tabulation they were grouped into sets fixed after 7–10, 12–13, and 16–19 days.

After fixation the cultures were dehydrated, embedded in paraffin and serially sectioned at 6 $\mu$. The sections were stained with haematoxylin-eosin, with Schiff's periodic acid method and with carmalum-orange G, anilin-blue (modified Azan).

### RESULTS

### *Control Explants*

The growth of the untreated lung explants has been described previously (Lasnitzki, 1956) and will only be shortly summarised here. Living explants become surrounded by an outgrowth. of translucent branching bronchioli while isolated fibroblasts and macrophages wander out from the explant towards the periphery of the clot. Histological examination of serial sections shows formation of new bronchioli at the periphery as well as in the centre of the explant. They are embedded in cellular connective tissue and lined with one row of cuboidal or cylindrical secretory epithelium (Fig. 1 and 2). Bronchial cartilage develops in many cultures : it is first discerned as a focus of condensed mesenchymal cells which after two to three weeks' cultivation differentiate into typical cartilage.

### *Effects of Smoke Condensates*

All four subfractions promoted the growth of the bronchiolar epithelium (Table I) but the degree, extent, induction time and the histological type of the hyperplasia varied considerably with the different fractions used.

Two main types of changes could be distinguished : generalised increase in formation of bronchioli was seen after short periods of treatment. The newly formed bronchioli were still lined with one row of cells but these were frequently

TABLE I.—*The Number of Treated Cultures Showing Growth Promotion and Hyperplasia*

| Time of treatment (days) | Whole neutral fraction: Denicotea cond. | Whole neutral fraction: Smoking machine cond. | Subfraction containing mainly hydrocarbons | Hydrocarbon-free subfraction |
|---|---|---|---|---|
| 7–10 | 2/9* | 7/13 | 13/18 | — |
| 12–13 | 4/16 | 2/8 | 6/10++ | 2/7 |
| 16–19 | 7/23+− | 5/8+ | 5/14+ | 12/22++ |

* The right-hand figures give the total number of cultures fixed at each point of observation.
+− Slight hyperplasia.
+ Moderate hyperplasia.
++ Extensive hyperplasia.

increased in height, particularly in bronchioli occupying the periphery of the explant (Fig. 3); hyperplasia of the bronchiolar epithelium occurred usually after the longer times of application and was more often seen in the larger bronchioli. The increased cell proliferation progressed from the periphery towards the centre of the bronchus and often led to partial or complete occlusion of the lumen.

The epithelial hyperplasia was of three different histological types: (1) basal cell hyperplasia in which small crowded basal cells were lined by an intact inner layer of columnar epithelium which was secreting considerably more than that in the controls; (2) loss of the secretory epithelium and pleomorphism of cells with irregular enlargement of the nuclei; (3) squamous metaplasia, also with loss of the secretory epithelium which was replaced by flattened cells with their long axes parallel to the lumen. The epithelium resembled the epidermis, except that there was no keratin formation.

*Effects of whole neutral fraction*

(a) *Denicotea condensate.*—This substance was only mildly active; after 7–10 days' treatment 2 out of 9 explants showed increased formation of new bronchi, after 12–13 days 4 out of 16 cultures exhibited similar changes while after 16–19 days in 7 out of 16 cultures a small number of bronchioli with basal cell hyperplasia were observed.

(*b*) *Smoking machine condensate.*—This was more effective than the Denicotea condensate. After 7–10 days' exposure, 9 out of 13 cultures showed increased bronchus formation and in 2 of these hyperplastic bronchioli were found as well. After 16–19 days' treatment, 5 out of 8 cultures showed both changes: i.e. more newly formed bronchi as well as increased proliferation of the lining epithelium. The hyperplastic epithelium consisted mainly of irregularly enlarged crowded cells with hyperchromatic nuclei; the secretory cells were lost (Fig. 4) and in a few bronchi there was a transition to squamous metaplasia.

*Neutral fraction containing mainly hydrocarbons.*—After 7–10 days' treatment 12 out of 18 cultures showed increased formation of new bronchi. After 12–13 days both changes, a generalised increase in new bronchioli as well as extensive basal cell hyperplasia of individual bronchioli were present in 6 out of 10 cultures. The hyperplastic epithelium consisted of 6–12 rows of densely crowded basal cells with abundant mitosis and was lined with intact functioning, secretory epithelium (Fig. 5, 6). After 16–19 days' treatment 4 out of 14 explants showed hyperplasia of a moderate degree.

*Neutral fraction without hydrocarbons.*—In this set of experiments the cultures were fixed after 12 and 19 days's exposure. After the shorter growth period 2 out of 7 explants showed increased bronchus formation, but after 19 days marked hyperplasia of the larger bronchioli was present in 12 out of 22 treated cultures. In these the epithelium had multiplied to 6–10 rows of cells which became stratified and had undergone squamous metaplasia (Fig. 7, 8).

### DISCUSSION

Since a statistical relationship between the increase of human lung cancer and smoking was first established (Doll and Hill, 1954, 1956; Hammond and Horn, 1954) many investigators have attempted to find experimental evidence for carcinogenic properties of cigarette smoke.

Cooper and Lindsey (1955) demonstrated the presence of small amounts of 3–4-benzpyrene and 1–12-benzperylene in the neutral fraction of cigarette smoke condensate. These findings were confirmed by Wright and Wynder (1955), Lettré, Jahn and Hausbeck (1956), and Bonnet and Neukomm (1957) who in addition isolated two other carcinogenic hydrocarbons, 1·2-benzanthracene and 3–4–9–10-dibenzpyrene from the neutral fraction.

Of the many workers who tried to induce animal tumours by application of cigarette smoke in various forms, Wynder, Graham and Croninger (1953, 1955) and Wynder, Kopf and Ziegler (1957) reported the production of skin carcinomas in various strains of mice painted for long periods with concentrated condensate, and Graham, Croninger and Wynder (1957) obtained cancer in rabbits' ears treated similarly. Blacklock (1957) produced one carcinoma and one sarcoma in the lungs of rats injected with a mixture of Denicotea condensate and killed tubercle bacilli. In all three experiments the induction period corresponded to over half the natural life span of the animals while the incidence of carcinomas varied from 3 to 44 per cent.

Gellhorn (1958) found that a combination of 3–4-benzpyrene and smoke condensate produced significantly more skin carcinomas in mice than either compound alone, a result which suggests either an additive effect due to the carcinogenic substances contained in smoke or a cocarcinogenic action.

Rockey *et al.* (1958) showed that tobacco tar applied to the bronchial mucosa of dogs induced hyperplasia and squamous metaplasia within a few weeks but there was no increased mitotic activity and no atypism. Leuchtenberger, Leuchtenberger and Doolin (1958) also observed hyperplasia and squamous metaplasia of the bronchial epithelium of mice exposed to cigarette smoke ; the hyperplastic epithelium was occasionally atypical and showed graded increases in nuclear volume, dry mass and DNA.

In the present experiments smoke condensate added to the medium of growing human foetal lung promotes the growth of the bronchiolar epithelium after an exposure of 10 to 19 days. At first a generalised increase in the formation of new bronchioli is observed ; this is followed by hyperplasia of the lining epithelium in individual bronchioli. The number of cultures affected, the extent of hyperplasia in them and its histological type varies with the different fractions used.

The early generalised growth-promotion is of the same histological type with all three fractions while the second stage—the hyperplasia of individual bronchioli—can be divided into three distinctive histological patterns : (1) basal cell hyperplasia with preservation of functioning secretory epithelium occurs after administration of the hydrocarbon fraction ; (2) loss of the secretory epithelium, irregular enlargement and hyperchromatosis of nuclei, is seen frequently after the whole fraction from smoking-machine condensate, while (3) squamous metaplasia also with loss of the secretory epithelium, is observed after treatment with the hydrocarbon-free fraction. Mitotic abnormalities are seen after all three fractions but are most frequent after the smoking machine condensate.

The epithelial hyperplasia resembles that observed after treatment with 20-methylcholanthrene in organ cultures of the mouse prostate and 3-4-benzpyrene in human foetal lung (Lasnitzki, 1951, 1956) except that the latter substance only rarely caused squamous metaplasia.

The identification of 3–4-benzpyrene in the neutral fraction of smoke condensate has focused attention on this compound as the causative agent in the carcinogenesis of lung tumours. Previous work has shown that the minimum dose of 3–4-benzpyrene necessary to induce epithelial hyperplasia of the bronchial epithelium under identical experimental conditions was 1 μg./ml. of medium applied for 4 weeks. In the present experiment the concentration used per ml. of medium was the yield of 0·2 cigarettes containing according to Cooper's and Lindsey's data (1955) $4^{-3}$ μg. of 3–4-benzpyrene, an amount far too low to account for the changes seen. It must be assumed therefore that they are due, at least as far as the hydrocarbon fraction is concerned, to the sum of all carcinogenic hydrocarbons present in the neutral fraction.

The fact that the non-hydrocarbons also stimulate the growth of the bronchial epithelium suggests that in addition to the hydrocarbons other compounds must play a rôle, either as causative or promoting agents.

Whether the hyperplasia induced experimentally *in vitro* would ultimately lead to true malignancy under the more complex conditions *in vivo* is not certain, but the results can nevertheless be considered a useful guide and suggest that the neutral fraction from smoke condensate may be carcinogenic to the human lung.

### SUMMARY

The effect of condensates from cigarette smoke was studied on organ cultures of human foetal lung.

Four different fractions were used : the whole neutral fraction from Denicotea condensate, the whole neutral fraction from a smoking machine, and two sub-fractions of the neutral Denicotea fraction, one containing mainly hydrocarbons, the other without hydrocarbons.

Control explants showed outgrowth of branching bronchioli lined with one row of cuboidal or cylindrical secretory epithelium.

All four condensates increased the formation of new bronchioli and induced hyperplasia of the lining epithelium in individual bronchioli leading to partial or complete occlusion of the lumen.

The changes were least marked after the whole neutral fraction from Denicotea condensate and most extensive after the hydrocarbon fraction.

The hyperplastic epithelium showed three different histological types depending on the fractions used.

Basal cell hyperplasia was seen after the whole neutral fraction from Denicotea condensate and after the hydrocarbon fraction, pleomorphism of cells with loss of the secretory epithelium was present after the neutral smoking machine fraction, while squamous metaplasia was observed after the hydrocarbon free fraction.

The changes resemble those obtained after treatment with 20-methylcholanthrene and 3–4-benzpyrene in organ cultures of the mouse prostate and human foetal lung, except that the latter substance rarely caused squamous metaplasia.

The very low amount of 3–4-benzpyrene present in the neutral fraction together with the finding that the hydrocarbon free fraction also produces hyperplasia indicates that the effects cannot be due to 3–4-benzpyrene alone.

I am indebted to Mr. Oswald Lloyd, F.R.C.S., and Dr. Bruce Eton of Addenbrooke's Hospital, Cambridge for their friendly co-operation in providing the foetuses used in these experiments. I would also like to thank Dr. Honor B. Fell, F.R.S. for advice and criticism in the preparation of this manuscript, Mr. George

Lenney, A.I.S.T., who made the microphotographs and Mrs. Marion Thomson for skilled technical assistance.

### REFERENCES

Blacklock, J. W. S.—(1957) *Brit. J. Cancer*, **11**, 181.
Bonnet, J. and Neukomm, S.—(1957) *Oncologia*, **10**, 125.
Cooper, R. L. and Lindsey, A. J.—(1955) *Brit. J. Cancer*, **9**, 304.
Doll, R. and Hill, A. B.—(1954) *Brit. med. J.*, i, 1451.—(1956) *Ibid.*, ii, 1071.
Fell, H. B. and Robison, R.—(1930) *Biochem. J.*, **24**, 1905.
Gellhorn, A.—(1958) *Cancer Res.*, **18**, 510.
Graham, E. A., Croninger, A. B. and Wynder, E. L.—(1957) *Ibid.*, **17**, 1058.
Hammond, E. C. and Horn, D.—(1954) *J. Amer. med. Ass.*, **155**, 1316.
Lasnitzki, I.—(1951) *Brit. J. Cancer*, **5**, 345.—(1956) *Ibid.*, **10**, 510.
Lettré, H., Jahn, A. and Hausbeck, Ch.—(1956) *Angew. Chem.*, **6**, 212.
Leuchtenberger, C., Leuchtenberger, R. and Doolin, P.—(1958) *Cancer*, **11**, 490.
Rockey, E. E., Kuschner, M., Kosak, A. I. and Mayer, E.—(1958) *Ibid.*, **11**, 466.
Wright, G. and Wynder, E. L.—(1955) *Proc. Amer. Ass. Cancer Res.*, **2**, 55.
Wynder, E. L., Graham, E. A. and Croninger, A. B.—(1953) *Cancer Res.*, **13**, 855.—(1955) *Ibid.*, **15**, 445.
Wynder, E. L., Kopf, P. and Ziegler, H.—(1957) *Cancer*, **10**, 1193.

---

### EXPLANATION OF PLATES

Fig. 1.—Section through a control culture after two weeks' growth *in vitro*, showing bronchioli embedded in cellular connective tissue. Haematoxylin-eosin. × 97.

Fig. 2.—Section through another control culture after the same growth period *in vitro*. The bronchioli are lined with one row of cuboidal or cylindrical secretory epithelium. Modified Azan. × 280.

Fig. 3.—Section through a culture grown for 10 days with the hydrocarbon fraction showing an increased number of bronchioli and enlargement of epithelial cells in some bronchioli. Periodic acid Schiff. × 97.

Fig. 4.—Section through a culture grown for 16 days with smoking machine condensate, showing hyperplasia of the bronchial epithelium. Note loss of secretory epithelium and irregularity of nuclear size. Haematoxylin-eosin. × 420.

Fig. 5.—Section through a culture grown for 13 days with the hydrocarbon fraction, showing marked hyperplasia of the basal cell type. Periodic acid Schiff. × 80.

Fig. 6.—Part of the same culture at higher magnification. Note abundance of mitosis and secretion. Periodic acid Schiff. × 230.

Fig. 7.—Section through a culture grown for 19 days with the hydrocarbon-free fraction. Haematoxylin-eosin. × 160.

Fig. 8.—Section through another culture treated for 19 days with the hydrocarbon-free fraction. Note transition to squamous metaplasia and loss of secretory epithelium. Haematoxylin-eosin. × 240.